# Long-Term Effects and Prognosis in Acute Heart Failure Treated with Tolvaptan: The AVCMA Trial

**DOI:** 10.1155/2014/704289

**Published:** 2014-11-10

**Authors:** Satoshi Suzuki, Akiomi Yoshihisa, Takayoshi Yamaki, Koichi Sugimoto, Hiroyuki Kunii, Kazuhiko Nakazato, Yukihiko Abe, Tomiyoshi Saito, Takayuki Ohwada, Hitoshi Suzuki, Shu-ichi Saitoh, Isao Kubota, Yasuchika Takeishi

**Affiliations:** ^1^Department of Cardiology and Hematology, Fukushima Medical University, 1 Hikarigaoka, Fukushima 960-1295, Japan; ^2^Department of Cardiology, Ohara Medical Center, Fukushima 960-0195, Japan; ^3^Second Department of Medicine, Shirakawa Kosei General Hospital, Shirakawa 961-0005, Japan; ^4^Department of Cardiology, Fukushima Red Cross Hospital, Fukushima 960-8530, Japan; ^5^First Department of Internal Medicine, Yamagata University School of Medicine, Yamagata 990-9585, Japan

## Abstract

*Background.* Diuresis is a major therapy for the reduction of congestive symptoms in acute decompensated heart failure (ADHF) patients. We previously reported the efficacy and safety of tolvaptan compared to carperitide in hospitalized patients with ADHF. There were some reports of cardio- and renal-protective effects in carperitide; therefore, the purpose of this study was to compare the long-term effects of tolvaptan and carperitide on cardiorenal function and prognosis. *Methods and Results.* One hundred and five ADHF patients treated with either tolvaptan or carperitide were followed after hospital discharge. Levels of plasma B-type natriuretic peptide, serum sodium, potassium, creatinine, and estimated glomerular filtration rate were measured before administration of tolvaptan or carperitide at baseline, the time of discharge, and one year after discharge. These data between tolvaptan and carperitide groups were not different one year after discharge. Kaplan-Meier survival curves demonstrated that the event-free rate regarding all events, cardiac events, all cause deaths, and rehospitalization due to worsening heart failure was not significantly different between tolvaptan and carperitide groups. *Conclusions.* We demonstrated that tolvaptan had similar effects on cardiac and renal function and led to a similar prognosis in the long term, compared to carperitide.

## 1. Introduction

Acute decompensated heart failure (ADHF) is detected by typical findings of low cardiac output associated with signs of pulmonary and systemic congestion [1–3]. Diuretics are the first line of treatment for ADHF, and The Acute Decompensated Heart Failure National Registry (ADHERE) has demonstrated that approximately 90% of patients hospitalized with ADHF receive intravenous loop diuretics [4]. However, some of the most important and potentially fatal disadvantages of diuretic therapy for ADHF patients are electrolyte abnormalities such as hyponatremia and worsening of renal function, which are both associated with longer hospital stays and higher mortality [5–8].

Intravenous administration of carperitide has been used as an acute phase therapy for ADHF due to its natriuretic and vasodilation effects, especially a high rate use (58.2%) in Japan [9, 10]. Several reports revealed the efficacy and safety of carperitide in acute phase treatment and the improvement of the long-term prognosis [11, 12], but the evidence is insufficient. Tolvaptan is a selective vasopressin V_2_ receptor antagonist that produces water excretion without changes in renal hemodynamics or electrolyte excretion [13]. In ADHF patients, oral tolvaptan in addition to standard therapy, including conventional diuretics, improved heart failure signs and symptoms without serious adverse events [14]. We previously reported the efficacy and safety of tolvaptan compared with carperitide in the acute heart failure volume control multicenter randomized (AVCMA) trial. In this study, tolvaptan brought about greater daily urine volume and less blood pressure reduction than carperitide in ADHF patients [15]. Moreover, it was demonstrated that the serum sodium level during administration of tolvaptan was higher than that of carperitide and that cardiovascular adverse events were less in the tolvaptan group than in the carperitide group during hospitalization [15]. Recently, a multicenter randomized control study reported that the long-term prognosis of low-dose carperitide in ADHF was improved compared to control group [12]. Therefore, the purpose of this study was to compare the long-term effects of tolvaptan on cardiorenal function and prognosis with those of carperitide.

## 2. Methods

### 2.1. Study Design

**Figure 1:**
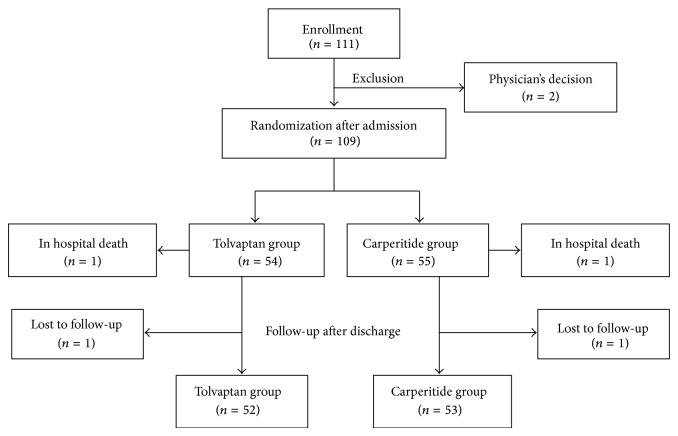
Participation flowchart.

AVCMA was a multicenter, randomized study, registered in the University Hospital Medical Information Network (ID, 000006258) as reported previously [15]. Written informed consent was obtained from all study subjects. The study protocol was approved by the ethical committee at the Fukushima Medical University and each participating institution, in compliance with the Declaration of Helsinki. Participants' flowchart in this study was shown in Figure 1. Sample size estimation of the AVCMA study was reported previously [15].

### 2.2. Study Participants

Patients were eligible for enrollment if they had presented volume fluid retention with ADHF or acute exacerbation of chronic heart failure (CHF), were diagnosed on the basis of the presence of at least one subjective symptom (dyspnea, orthopnea, or leg edema), and had one sign (rales, peripheral edema, ascites, or pulmonary vascular congestion on chest X-ray) of heart failure. Patients with acute myocardial infarction, severe hypotension (cardiogenic shock), anuria, hypernatremia (Na > 147 mEq/L), or who did not feel thirsty or have difficult water intake, were excluded. These ADHF patients underwent the standard initial treatment, including conventional loop diuretic administration, and were randomly assigned into two groups: oral administration of tolvaptan or continuous intravenous infusion of carperitide. Tolvaptan was orally administered at 3.75–15 mg per day, and carperitide was administered at 0.0125–0.025 *μ*g/kg depending on the pathological condition of the patient. Study subjects underwent blood sample examination, and levels of plasma B-type natriuretic peptide (BNP), serum sodium, potassium, creatinine, and estimated glomerular filtration rate (eGFR) were measured before administration of tolvaptan or carperitide at baseline, the time of discharge, and one year after discharge. GFR was estimated from the modification of diet in renal disease formula for the Japanese.

One hundred and nine patients were enrolled in the AVCMA study [15], and these were randomly assigned to two groups (54 in the tolvaptan group and 55 in the carperitide group). Two patients died during the first admission (one with pneumonia and one with cancer after observation period of the AVCMA study). All patients were followed-up for almost one year after discharge (mean 296 days, 14–400 days), and two patients could not be followed up. Therefore, 105 subjects were analyzed in this study (52 in the tolvaptan group and 53 in the carperitide group) as shown in Figure 1. As shown in Table 1, baseline clinical characteristics on admission were not different between the tolvaptan and carperitide groups. There were no differences in medications at discharge between two groups (Table 2). The endpoints, which were judged independently by researchers, were (1) all cause death, (2) cardiac death, defined as death from worsening heart failure or sudden cardiac death, and (3)rehospitalization due to worsening heart failure.

### 2.3. Statistical Analysis

Results are expressed as mean ± standard deviation (SD), and skewed variables are presented as median and interquartile range. A *P* value of 0.05 was considered statistically significant, but no adjustment was made for multiplicity. Significance between the 2 groups was determined by unpaired Student's *t*-test for continuous variables and by Chi-square test for discrete variables. The changes in blood sample data from baseline within the same group were determined by paired *t*-test. If data were not distributed normally, the Mann-Whitney *U* test was used. Missing data were excluded from the analysis. Kaplan-Meier survival curves determined the time-dependent cumulative cardiac event-free rates in patients stratified between tolvaptan and carperitide groups and were analyzed by a log rank test. Statistical analysis was performed with a standard statistical program package (SPSS version 21.0, IBM, Armonk, NY, USA).

## 3. Results

### 3.1. Comparisons of Laboratory Data One Year after Discharge

**Figure 2:**
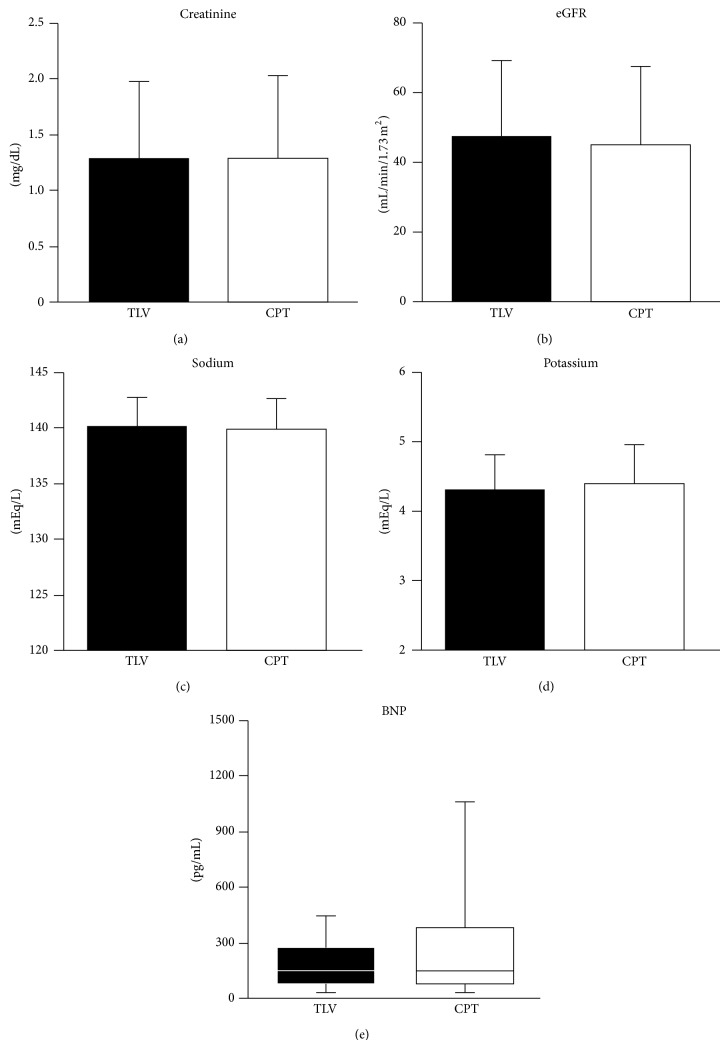
Comparisons of serum creatinine (a), estimated glomerular filtration rate (eGFR) (b), serum sodium (c), serum potassium (d), and plasma B-type natriuretic peptide (BNP) (e) at one year after initiating drug administration. TLV, tolvaptan group; CPT, carperitide group.

**Figure 3:**
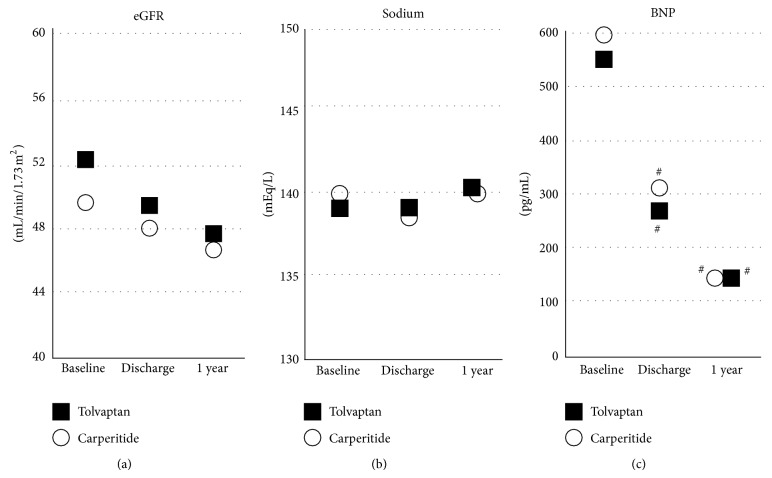
Comparisons of trends in eGFR (a), serum sodium level (b), and plasma BNP level (c) between tolvaptan (*n* = 52) and carperitide (*n* = 53) groups. ^#^ *P* < 0.05 versus baseline of same group. Mean values of eGFR and sodium and median values of BNP were plotted.

As shown in Figure 2, the levels of serum creatinine (a) and eGFR (b) one year after discharge were not significant between the tolvaptan group and carperitide group (serum creatinine: 1.29 ± 0.76 mg/dL in tolvaptan group versus 1.35 ± 0.98 mg/dL in carperitide group, *P* = 0.7634; eGFR: 47.7 ± 21.6 mL/min/1.73 m^2^ in tolvaptan group versus 44.9 ± 22.7 mL/min/1.73 m^2^ in carperitide group, *P* = 0.6085). These levels did not change one year after discharge compared to levels at discharge (Figure 3(a)).

The serum sodium (Figure 2(c)) and potassium (Figure 2(d)) levels were also not different between the tolvaptan group and carperitide group (serum sodium: 140.1 ± 2.9 mEq/L in tolvaptan group versus 139.5 ± 3.3 mEq/L in carperitide group, *P* = 0.3847; serum potassium: 4.32 ± 0.53 mEq/L in tolvaptan group versus 4.33 ± 0.65 mEq/L in carperitide group, *P* = 0.9263). The serum sodium level in tolvaptan and carperitide groups did not change at baseline, discharge, and one year after discharge (Figure 3(b)).

Plasma BNP levels in the tolvaptan group one year after discharge did not show a statistically significant difference compared to the carperitide group (147.5 (79.7–275.1) pg/mL versus 147.1 (74.8–385.0) pg/mL, *P* = 0.0915) (Figure 2(e)). Plasma BNP levels of both tolvaptan and carperitide groups one year after discharge were decreased from baseline but did not change compared to levels at discharge (Figure 3(c)).

### 3.2. Prognostic Analysis

**Figure 4:**
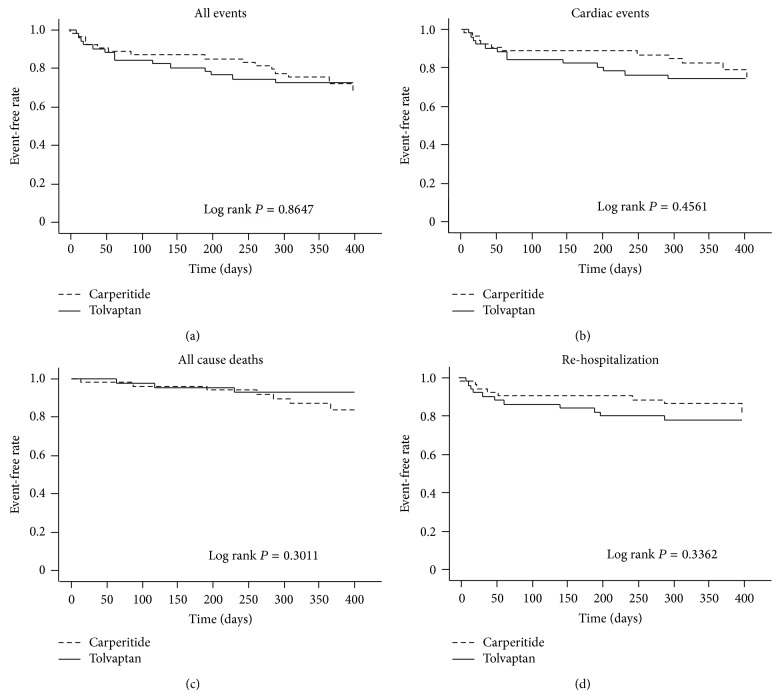
Kaplan-Meier analysis for all events (a), all cardiac events (b), all cause deaths (c), and rehospitalization (d) between tolvaptan group and carperitide group.

There were 29 cardiac events (10 all cause deaths including 5 cardiac deaths and 19 rehospitalization) during the follow-up period in all subjects. Cumulative event-free survival curves were illustrated by the Kaplan-Meier method and compared by a log rank test (Figure 4). Event-free rates regarding all events (a), cardiac events (b), all cause deaths (c), and rehospitalizations due to worsening heart failure (d) were not significantly different between the tolvaptan group and carperitide group (all events: 72.5% versus 72.2%, *P* = 0.8647; cardiac events: 74.5% versus 79.6%, *P* = 0.4561; all cause deaths: 94.1% versus 87.0%, *P* = 0.3011; rehospitalization due to worsening heart failure: 78.4% versus 85.1%, *P* = 0.3362) (Figure 4).

## 4. Discussion

The present study showed for the first time that there were no significant differences in renal function, electrolyte levels, plasma BNP levels, and prognosis from tolvaptan compared to carperitide after a long-term period of drug administration.

Diuretics are major therapeutic drugs for ADHF. In the ADHERE registry, it was reported that intravenous loop diuretics remain as the first-line therapy for ADHF and are currently prescribed for more than 90% of hospitalized ADHF patients [16]. In addition, carperitide, which develops vasodilation and natriuretic effects, is used frequently in Japan [9, 10]. A large-scale multicenter cohort study of more than 4,842 registered members (acute decompensated heart failure syndrome; ATTEND registry) revealed that although Japanese patient characteristics did not differ from those reported in Western countries, there was a unique finding of a high rate of carperitide use (58.2%) for ADHF in Japan [9, 10]. Tolvaptan is an orally administered active vasopressin V_2_ receptor antagonist that promotes aquaresis, and we therefore selected carperitide as a competitor to tolvaptan and compared the diuretic effects of the two.

The efficacy and safety of carperitide in the acute phase treatment of ADHF were reported by the Carperitide Effects Observed Through Monitoring Dyspnea in Acute Decompensated Heart Failure Study (COMPASS) trial in 2008 [11]. Also, a long-term prognostic examination of carperitide revealed that it led to a significant reduction of cardiac events compared to standard therapy without carperitide [12]. We previously reported that tolvaptan induced less reduction of blood pressure and cardiovascular events despite inducing higher urine volume than carperitide [15]. Then we evaluated the long-term prognostic effect of tolvaptan compared to carperitide as a follow-up examination of the AVCMA trial. As shown in this report, adverse events over a long-term period were not significantly different between the tolvaptan group and carperitide group.

Renal dysfunction is a frequent finding in patients with ADHF and is a powerful independent prognostic factor for prolonged length of hospital stay, increased in-hospital mortality, and higher rates of rehospitalization and death after discharge [17–19]. Several reports revealed that the use of a high dose of diuretics was a prognostic factor of worsening renal function and prognosis of ADHF patients [7, 17, 20], and inhibiting worsening renal function is an important issue for ADHF patients. Several reports revealed the renal protective effect of carperitide in the acute phase [11, 21]. Our previous report revealed that renal function estimated by serum creatinine and eGFR was not significantly different between tolvaptan and carperitide groups and that the incidence of worsening renal function (serum creatinine > 0.3 mg/dL increase during treatment) was also not different between these two groups [15]. In this report, renal function one year after treatment was also unchanged between these two groups. Moreover, the cardioprotective effect of carperitide was reported in basic and clinical examination [22, 23], and our present study demonstrated that plasma BNP level was not statistically significant but tended to be lower in the tolvaptan group than in the carperitide group one year after drug administration. This suggests that tolvaptan did not adversely affect renal and cardiac functions in ADHF patients than carperitide one year after starting drug administration.

### 4.1. Study Limitations

First, tolvaptan is different from carperitide in terms of administration route (tolvaptan is orally administered and carperitide intravenously). In the present study, enrollment-eligible patients did not have any severe hemodynamic abnormalities requiring an auxiliary circulation system such as an intra-aortic balloon pump or percutaneous cardiopulmonary support, because they often had difficulty with sufficient oral intake and complaint of thirst. There were also no patients who needed ventilation. If the PROTECT study regarding long-term prognostic analysis of carperitide was conducted with enrolled NYHA III or IV patients [12], then the characteristics of study participants might have been different in this study. Second, some patients were continuously administered tolvaptan after discharge, but outpatients enrolled in the carperitide group were not administered carperitide after discharge unless there was a rehospitalization. There is a possibility of greater effectiveness of tolvaptan in outpatients who were continuously administered the drug. Third, the mechanism of this study's results was not clear. Fourth, the number of study subjects was small. In future research, a larger scale study will be necessary to validate the true clinical usefulness of tolvaptan in Japan.

## Figures and Tables

**Table 1:** Comparisons of clinical characteristics between tolvaptan and carperitide groups.

	Tolvaptan	Carperitide
	(*n* = 52)	(*n* = 53)
Age (years)	74.6 ± 11.4	75.1 ± 10.4
Gender (male/female)	28/24	31/22
Body weight (kg)	58.2 ± 12.9	57.0 ± 11.7
Body mass index (kg/m^2^)	24.0 ± 4.3	22.5 ± 3.1
Etiology of chronic heart failure, *n* (%)		
Dilated cardiomyopathy	21 (40)	22 (42)
Ischemic heart disease	13 (25)	13 (25)
Valvular heart disease	6 (12)	5 (9)
Hypertensive heart disease	5 (10)	6 (11)
Other	7 (13)	7 (13)
Heart rate (/min)	90.0 ± 25.6	86.7 ± 25.9
Systolic blood pressure (mmHg)	130.0 ± 25.8	129.1 ± 26.5
Diastolic blood pressure (mmHg)	75.5 ± 15.4	73.9 ± 18.4
Echocardiography		
LVEDD (mm)	52.4 ± 10.7	53.0 ± 8.8
LVEF (%)	46.7 ± 17.1	44.7 ± 14.2
IVC (mm)	19.4 ± 5.7	18.7 ± 5.6
BNP^*^ (pg/mL)	544.3 (410.7)	599.0 (400.9)
Blood urea nitrogen (mg/dL)	24.4 ± 14.8	24.5 ± 12.6
Serum creatinine (mg/dL)	1.19 ± 0.75	1.23 ± 0.75
Estimated GFR (mL/min/1.73 m^2^)	52.4 ± 21.8	50.0 ± 21.2
Serum sodium (mEq/L)	139.7 ± 5.4	140.2 ± 3.5
Serum potassium (mEq/L)	4.1 ± 0.6	4.2 ± 0.6

LVEDD, left ventricular end diastolic diameter; LVEF, left ventricular ejection fraction; BNP, B-type natriuretic peptide; GFR, glomerular filtration rate.

^*^Skewed data are reported as median (interquartile range).

**Table 2:** Comparisons of medications between the tolvaptan and carperitide groups at discharge.

	Tolvaptan	Carperitide
	(*n* = 52)	(*n* = 53)
Loop diuretics, *n* (%)	43 (83)	44 (83)
Thiazide diuretics, *n* (%)	4 (8)	4 (7)
Spironolactone, *n* (%)	29 (56)	31 (58)
*β*-Blocker, *n* (%)	29 (56)	33 (62)
ACE inhibitors or ARBs, *n* (%)	26 (49)	32 (60)
Ca-Blocker, *n* (%)	9 (17)	6 (11)

ACE, angiotensin converting enzyme; ARB, angiotensin receptor blocker.
